# Using Slit-Lamp Images for Deep Learning-Based Identification of Bacterial and Fungal Keratitis: Model Development and Validation with Different Convolutional Neural Networks

**DOI:** 10.3390/diagnostics11071246

**Published:** 2021-07-12

**Authors:** Ning Hung, Andy Kuan-Yu Shih, Chihung Lin, Ming-Tse Kuo, Yih-Shiou Hwang, Wei-Chi Wu, Chang-Fu Kuo, Eugene Yu-Chuan Kang, Ching-Hsi Hsiao

**Affiliations:** 1 Department of Ophthalmology, Chang Gung Memorial Hospital, Linkou Medical Center, No. 5 Fu-Hsin Rd, Kweishan, Taoyuan 333, Taiwan; shsk1212@gmail.com (N.H.); yihshiou.hwang@gmail.com (Y.-S.H.); weichi666@gmail.com (W.-C.W.); 2 College of Medicine, Chang Gung University, No. 261, Wenhua 1st Rd., Kweishan, Taoyuan 333, Taiwan; 3 Center for Artificial Intelligence in Medicine, Chang Gung Memorial Hospital, Linkou Medical Center, No. 5 Fu-Hsin Rd, Kweishan, Taoyuan 333, Taiwan; asign1022@gmail.com (A.K.-Y.S.); lin3031@gmail.com (C.L.); zandis@gmail.com (C.-F.K.); 4 Department of Ophthalmology, Kaohsiung Chang Gung Memorial Hospital, No. 123, Dapi Rd, Niaosong, Kaohsiung 833, Taiwan; mingtse@cgmh.org.tw

**Keywords:** deep learning, infectious keratitis, cropped corneal image, slit-lamp images

## Abstract

In this study, we aimed to develop a deep learning model for identifying bacterial keratitis (BK) and fungal keratitis (FK) by using slit-lamp images. We retrospectively collected slit-lamp images of patients with culture-proven microbial keratitis between 1 January 2010 and 31 December 2019 from two medical centers in Taiwan. We constructed a deep learning algorithm consisting of a segmentation model for cropping cornea images and a classification model that applies different convolutional neural networks (CNNs) to differentiate between FK and BK. The CNNs included DenseNet121, DenseNet161, DenseNet169, DenseNet201, EfficientNetB3, InceptionV3, ResNet101, and ResNet50. The model performance was evaluated and presented as the area under the curve (AUC) of the receiver operating characteristic curves. A gradient-weighted class activation mapping technique was used to plot the heat map of the model. By using 1330 images from 580 patients, the deep learning algorithm achieved the highest average accuracy of 80.0%. Using different CNNs, the diagnostic accuracy for BK ranged from 79.6% to 95.9%, and that for FK ranged from 26.3% to 65.8%. The CNN of DenseNet161 showed the best model performance, with an AUC of 0.85 for both BK and FK. The heat maps revealed that the model was able to identify the corneal infiltrations. The model showed a better diagnostic accuracy than the previously reported diagnostic performance of both general ophthalmologists and corneal specialists.

## 1. Introduction

Microbial keratitis (MK) is a serious corneal disease that can lead to reduced vision and even blindness [1,2]. The annual incidence of MK as a cause of monocular blindness ranges from 1.5 to 2 million cases worldwide [3]. It is considered an epidemic, particularly within South Asia, Southeast Asia, and East Asia, and in regions where fungal keratitis (FK) accounts for more than 50% of all MK cases [4]. The management of FK is challenging and may require surgical intervention, and FK has been reported to have poor visual outcomes [5,6]. Hence, early diagnosis is essential for avoiding devastating vision-threatening outcomes.

However, the early diagnosis of FK is also challenging. Although some predisposing factors and clinical features could lead ophthalmologists to suspect fungal infection [7], culture-based methods, which are laborious and time consuming, remain the cornerstone of diagnosis [8]. The time gap between patient presentation and diagnosis may lead the patient to miss the optimal time for treatment initiation, resulting in deep fungal invasion. Therefore, ophthalmologists may start antifungal medication based on distinctive lesions of each pathogenic microorganism identified on the cornea. Previous studies have reported that with an image-only diagnosis, general ophthalmologists are only able to correctly distinguish FK from bacterial keratitis (BK) 49.3–67.1% of the time [9,10], and this percentage ranges from 66.0% to 75.9% among corneal specialists [10,11].

Deep learning algorithms with artificial intelligence (AI) have demonstrated an exceptional performance at detecting various ocular conditions through different image modalities, such as fundus photos for diabetic retinopathy [12], age-related macular degeneration [13], retinal nerve fiber layer thickness and visual field for glaucoma [14], and topography for keratoconus [15]. AI appears to be a promising tool for first-line medical care, especially in scenarios in which ophthalmologists are not readily available. However, only limited studies have applied AI for the diagnosis of FK by using slit-lamp images, [9,10] and the accuracy was approximately 69% [10]. In this study, we aimed to develop a deep learning model that uses cropped slit-lamp images and to improve the prediction in differentiating BK and FK.

## 2. Materials and Methods

### 2.1. Identification of Microbial Keratitis

Patients with culture-proven MK who presented to Chang Gung Memorial Hospital, Linkou Medical Center, and Kaohsiung Medical Center in Taiwan between 1 January 2010 and 31 December 2019 were recruited. MK diagnosis was corroborated by the clinical manifestations of corneal infection and pathogen identification of the sample from the infection site. Corneal scrapings obtained from patients with presumed MK underwent smear and culture examinations for the detection of bacteria, mycobacteria, and fungi through the use of standard microbiological culture techniques [16,17], including blood and chocolate agar (Nippon Becton Dickinson Co. LTD, Akasaka Garden City, Japan), inhibitory mold agar (IMA) and IMA supplemented with chloramphenicol and gentamicin (Creative CMP^®^ New Taipei City, Taiwan), Lowenstein–Jensen agar slants (Creative CMP^®^ New Taipei City, Taiwan), and thioglycolate broth (BioStar, Taichung City, Taiwan). Cultures were defined as positive if microbial growth was observed on two media, microbial elements were observed in smears and growth on one medium, or confluent microbial growth was observed on one medium.

### 2.2. Exclusion Criteria

Patients were excluded if they had mixed bacterial and fungal infections; corneal perforation; no documented slit-lamp images; poor-quality or fluorescein-staining images; a history of ocular surface surgery, such as penetrating keratoplasty, and amniotic membrane transplantation; or the presence of other corneal diseases, such as viral keratitis, Acanthamoeba keratitis, marginal keratitis, corneal opacity, chemical burn, Stevens–Johnson syndrome, mucous membrane cicatricial pemphigoid, or bullous keratopathy.

### 2.3. Image Collection

We obtained slit-lamp images from two centers by using the same standard procedure certified by ophthalmic technicians. Images from Linkou Medical Center were captured with a Canon EOS 7D camera mounted on Haag-Streit BX900 slit-lamp microscopy, and images from Kaohsiung Medical Center were captured with a Nikon D100 camera mounted on a Topcon SL-D8 slit-lamp biomicroscope (before May 2015) and Canon EOS 7D camera mounted on a Haag-Streit BX900 slit-lamp microscope (after May 2015) [10]. Images with white light illumination without slit-beam enhancement from each patient were used for image classification.

### 2.4. Algorithm Architecture

**Figure 1:**
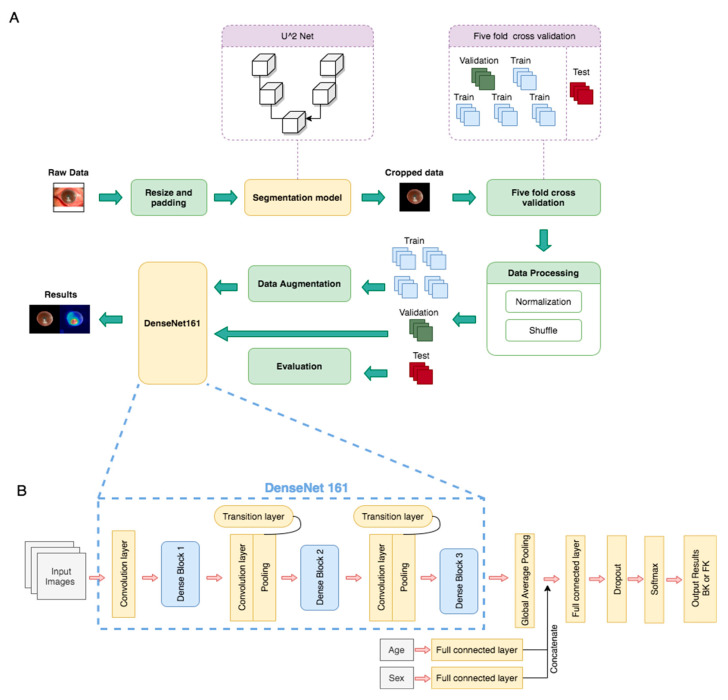
Deep learning framework for fungal keratitis diagnosis based on slit-lamp images. (**A**) Model architecture, including the segmentation model and classification model (DenseNet161). (**B**) Architecture of DenseNet161, which was the deep learning neural network selected as the basis for this study.

**Figure 2:**
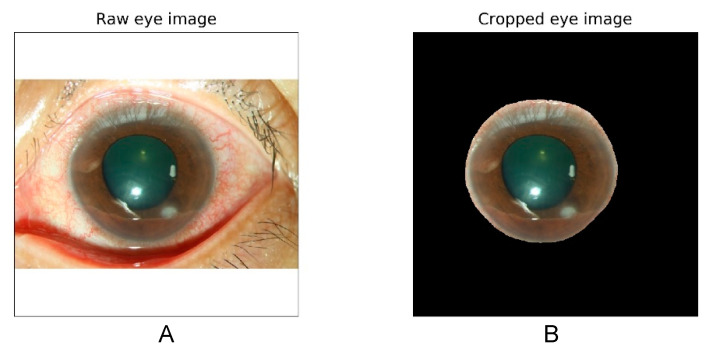
Raw slit-lamp images with white illumination (**A**) and cropped corneal images (**B**) excluding the eyelid and the conjunctival area.

The algorithm architecture is illustrated in Figure 1. The algorithm was divided into two main parts, namely the segmentation model and classification model. We trained the segmentation model by using U square Net (U^2^ Net) to crop the image of the cornea (sample in Figure 2). The U^2^ Net model performed better than the U-net and U-net++ models did because it could access more information and preserve complete features of the cornea [18]. All of the images were then resized to a resolution of 512 × 512 × 3 before being input into the U^2^ Net model. We also normalized each image into (0,1) and then augmented the image by subtracting 0.485 and dividing by 0.299, which enabled the model to converge more quickly and steadily. A total of 100 patients were randomly selected and divided into training, validation, and testing sets, which consisted of 70 patients (183 images), 20 patients (45 images), and 10 patients (15 images), respectively. The U^2^ Net Intersection over Union model achieved accuracies of 93% on the validation set and 95% on the independent testing set. Furthermore, the trained U^2^ Net model was applied to a total of 580 patients (1330 images).

In the classification model, we used five-fold cross-validation to divide the cropped images from the aforementioned U^2^ Net segmentation model. For data randomization, we set different seeds to simulate the same sequence of number randomization in each seed. We categorized the images in the data set according to the patient. The whole data set was then split into six parts; one-sixth of the data set was reserved for testing, and the remaining five parts were used for cross-validation training. For each patient, the data were separated into training, validation, and testing data sets at a ratio of 4:1:1. We also used image augmentation approaches including random brightness adjustment, saturation adjustment, contrast adjustment, horizontal flipping, rotation, and normalization, as shown in Supplementary Figure S1.

For the classification model, we applied various convolutional neural networks (CNNs), including ResNet50, ResNet101, DenseNet121, DenseNet161, DenseNet169, DenseNet201, InceptionV3, and EfficientNetB3. DenseNet161 was used as the basis of our classification model, as shown in Figure 1, with preweights from ImageNet Large Scale Visual Recognition Competition [19,20]. Age and sex were also input into fully connected layers and yielded two output vectors; the output vectors were then concatenated into the vectors produced from the global average pooling layer [21]. The model was trained up to 100 epochs and established on the basis of maximum accuracy and minimum loss in the validation set.

### 2.5. Performance Interpretation and Statistics

For visualizing heat maps, the gradient-weighted class activation mapping (Grad-CAM) technique [22], in which the model’s attention scores are computed according to the calculation of the gradients of the model’s output and the last convolutional layer, was used to plot the heat map of the model. Receiver operating characteristic (ROC) curves were illustrated to discriminate between BK and FK, and the area under the curve (AUC) was measured. From the ROC curve, Youden’s index was used to obtain the sensitivity and the specificity. The accuracy of the model was also calculated. Statistical analysis was performed with IBM SPSS Statistics Version 23 (SPSS, Inc., Chicago, IL, USA).

## 3. Results

### 3.1. Patient Classification and Characteristics

A total of 580 patients (420 male and 160 female) with 1330 images (with only one eye involved) were included. The average patient age was 55.4 years. According to the culture results, 346 patients (824 images) were classified as having BK and 234 patients (506 images) were classified as having FK. The final data set consisted of 388 patients (904 images) for training, 96 patients (212 images) for validation, and 96 patients (214 images) for testing. The distribution and characteristics of the patients are shown in Table 1.

### 3.2. Performance of Different Models

We evaluated the performance of the models by using the validation and testing data sets; the average accuracy was approximately 80%. Details regarding the accuracy, sensitivity, and specificity of all of the models are presented in Table 2. The rate of diagnostic accuracy for BK ranged from 79.6% to 95.9%, and that for FK ranged from 26.3% to 65.8%. DenseNets, EfficientNets, and InceptionV3 exhibited a similar performance; the average accuracy ranged from approximately 76% to 79%, and the diagnostic rates for FK were all between 56% and 66%. By contrast, ResNet achieved a diagnostic rate of over 90% for BK but below 50% for FK.

**Figure 3:**
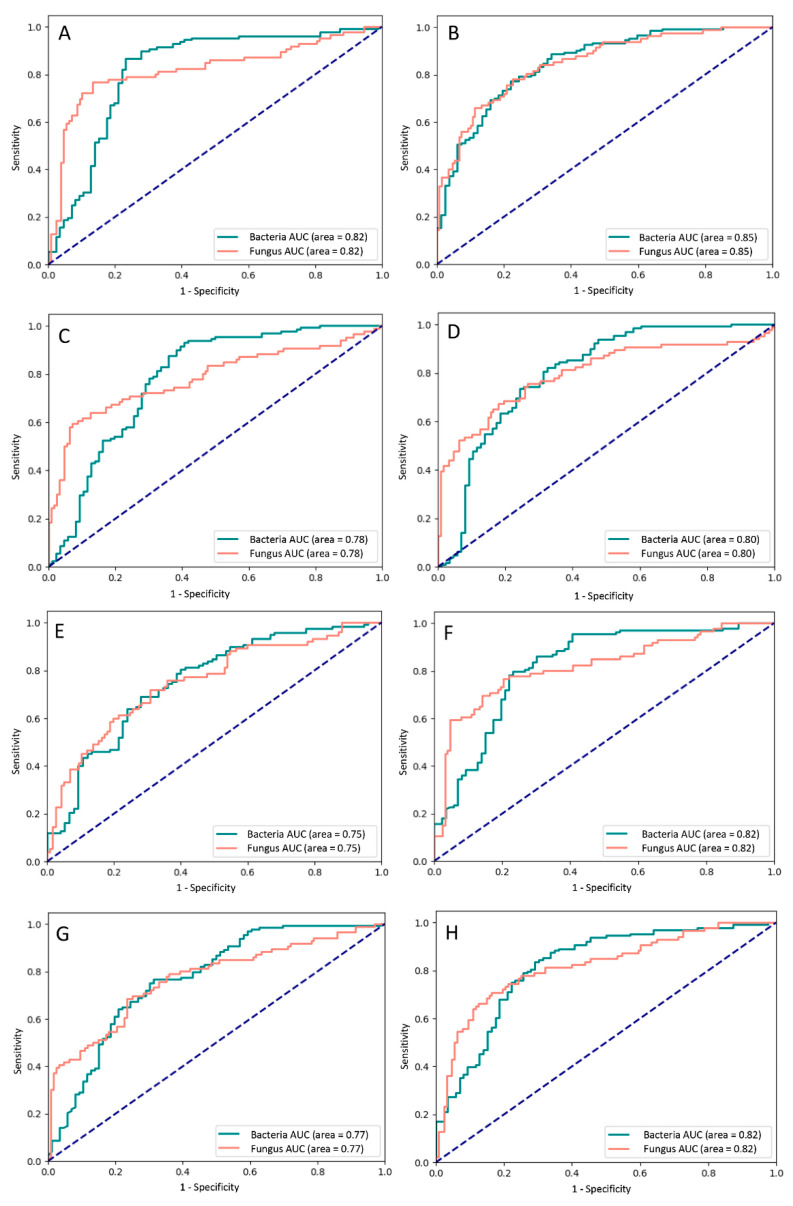
Receiver operating characteristic curves of the different deep learning models with (**A**) DenseNet121, (**B**) DenseNet161, (**C**) DenseNet169, (**D**) DenseNet201, (**E**) EfficientNetB3, (**F**) InceptionV3, (**G**) ResNet101, and (**H**) ResNet50. AUC—area under the curve.

**Figure 4:**
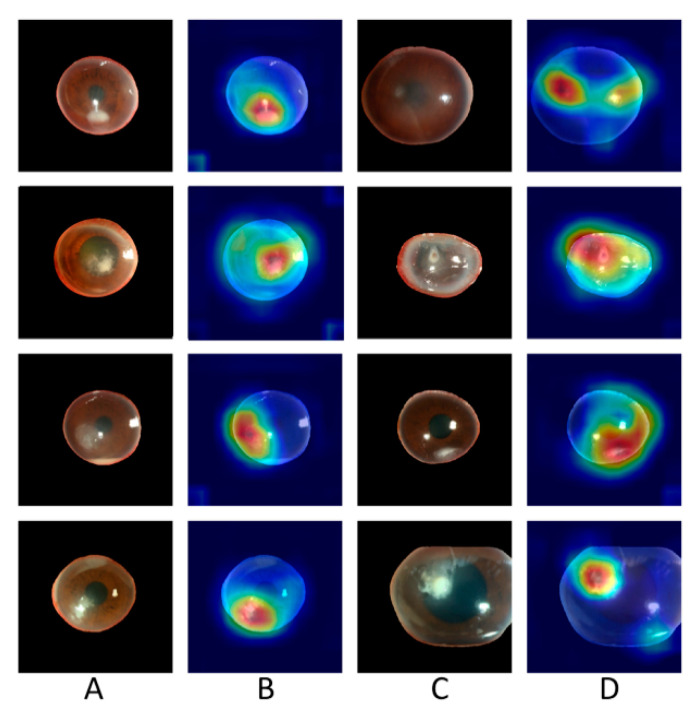
Sample heat maps generated by the model that correctly diagnosed patients as having bacterial keratitis and fungal keratitis. Column (**A**): images of fungal keratitis. Column (**B**): heat maps of the corresponding images in Column (**A**). Column (**C**): images of bacterial keratitis. Column (**D**): heat maps of the corresponding images in Column (**C**).

DenseNet161 achieved the best performance in the prediction of BK and FK, with an AUC of the ROC curve of 0.85 for both BK and FK (Figure 3). The diagnostic accuracy for BK was 87.3%, and that for FK was 65.8%. A sample heat map generated with Grad-CAM for model visualization is presented in Figure 4. With the cropped corneal images, the model was able to identify the corneal infiltrations and focus on the pathology of MK while ignoring most of the reflected light on the cornea.

## 4. Discussion

In this study, we developed a deep learning model to differentiate MK into BK and FK. The model using cropped slit-lamp images of the cornea with white light illumination achieved an AUC of 0.85. The accuracy approached 80%, exceeding that of general ophthalmologists and is comparable to that of corneal specialists [9,10].

Early diagnosis of FK is important but challenging. No pathognomonic features can wholly support a physician’s diagnosis, and delayed diagnosis can increase the difficulty of FK management, often necessitating surgical intervention and resulting in poor visual outcomes [5]. Culture-based methods are the current cornerstone for FK diagnosis; however, a time lag exists between patient presentation and diagnosis. Dalmon et al. reported that the clinical signs of BK and FK can be used to identify their causative organisms. In their study, 15 corneal specialists assessed 80 slit-lamp images and were able to correctly differentiate between BK and FK 66% of the time [11].

Few studies have applied AI and deep learning models for FK diagnosis by using slit-lamp images. Kuo et al. developed a slit-lamp image–based deep learning model for FK diagnosis. Although their work yielded promising results, the average reported accuracy was 69.4%, and the ROC curve was only 0.65; moreover, the diagnostic rate was lower than the rate reported by corneal specialists in their own study [10]. According to their findings, the wrong prediction of the model was due to incorrect focusing on the eyelid, eyelash, and sclera [10]. The use of fluorescein-staining images was reported in only one study; however, the researchers aimed to identify early corneal ulcers by recognizing point-like patterns, which could not differentiate fungal ulcers that were difficult to manage from other corneal ulcers [23]. Xu et al. reported diagnostic rates of 80% for MK, 53.3% for BK, and 83.3% for FK by using a deep sequential-level learning model with slit-beam slit-lamp images. The accuracy of their model exceeded that of ophthalmologists (49.3% ± 11.9%) for over 120 test images [9]. However, the model required sophisticated patch sampling over the cornea and the lesion, as well as the application of an additional sequential model, constituting a relatively complicated approach.

**Figure 5:**
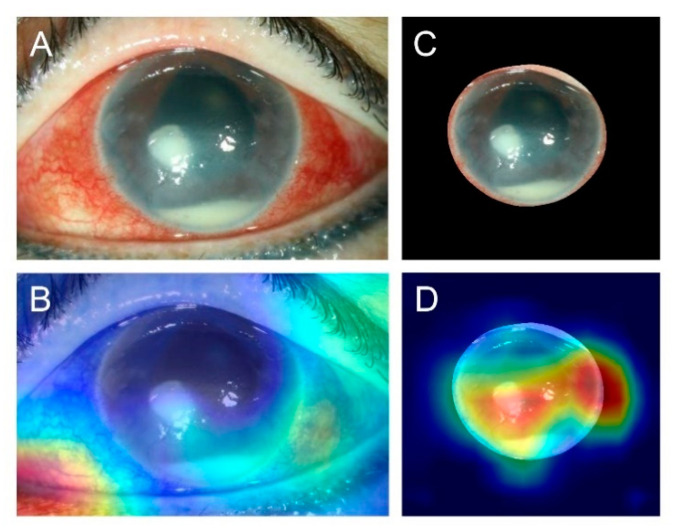
Sample images comparing slit-lamp with and without cropping. (**A**) Original slit-lamp image without cropping in an eye with bacterial keratitis. (**B**) Heat map generated by the model with the wrong prediction using the uncropped image showing the incorrect focus on the eyelid. (**C**) Cropped image from the slit-lamp image. (**D**) Heat map generated by the model with correct prediction using the cropped image showing the focus on corneal infiltration, corneal edema, and hypopyon.

In the present study, our model achieved an average accuracy of approximately 80% and diagnostic accuracies of approximately 80% and 60% for BK and FK, respectively (Table 2), and we used approximately 1.5-times more training images for BK than for FK. To alleviate the incorrect focusing reported in the previous study [10], we used cropped corneal images to train the model. We also evaluated the performance using slit-lamp images without cropping to train and test the model, but the average accuracy was decreased to approximately 70% (data not shown), which was comparable with the previous study [10]. The decreased performance may be due to inappropriate focusing on the area without clinically relevant features. (Figure 5).

The other previous model developed by Xu et al. achieved a higher model accuracy and a better diagnostic rate for FK than our model did; their overall accuracy was enhanced by the diagnostic accuracy for herpes simplex keratitis and other corneal diseases, which reached 93.3% and 90.0%, respectively. Furthermore, approximately 1.3-times more training images were used for FK than for BK. These results also indicate that the model accuracy was strongly influenced by the number of training images used, which supports our expectation that the integration of more images can help deep learning models become a robust tool for assisting early FK diagnosis [24].

We also tested various models by using different CNNs in our study. All of the models had an average accuracy between 76% and 80%. Because we applied five-fold cross-validation, both the validation and test data sets were independent of the training data set; thus, we also considered the validation accuracy and selected the best model based on the average validation and test performance. Among all of the models, DenseNet161 achieved the highest FK diagnostic accuracy (65.8%) and a relatively high average accuracy (78.6%). DenseNet outperformed other CNNs because of its unique architectural design, which efficiently took the features of every layer and reused them in all the subsequent layers, thereby enhancing the model [20]. In comparison, ResNet101 had the highest average diagnostic accuracy (80.0%) for BK and FK; however, its diagnostic rate for FK was relatively low (49.1%). The diagnostic accuracy of ResNet101 also had the largest standard deviation, indicating that the results varied considerably between layers.

From the heat maps generated with Grad-CAM, the model could identify the pathology of infection and ignore artifacts from reflected light by using cropped images of the cornea alone. We noticed that when the slit-lamp images were not cropped, the model focused on regions outside the cornea, such as the eyelid or conjunctiva. Therefore, we cropped the cornea by training a U^2^ segmentation model. To differentiate FK from BK, the model focused on the feature of corneal infiltration.

In this study, we demonstrated the promising role of AI in the diagnosis of infectious keratitis through the use of images only. Although microbiological culture remains the gold standard for FK diagnosis, early clinical detection of potential FK could aid the subsequent initiation of empirical treatment or referral management. Because most ophthalmologists and general practitioners may not have extensive experience in FK diagnosis, the deep learning-based model may help clinicians improve their diagnostic accuracy and subsequently initiate early and appropriate treatment. Moreover, AI can provide disease screening or telemedicine in places where prompt medical or ophthalmologic evaluation is infeasible.

This study has some limitations. First, we excluded patients with poor-quality slit-lamp images before training the model. However, poor-quality images are encountered in daily clinical practice, and the model performance may have thus been affected by factors such as patient cooperation, light reflection in the images, and photographer experience. Second, although corneal images are relatively easy to capture, environmental factors may render their real-world application challenging [25]. Third, fewer FK than BK cases and images were documented, which caused an imbalance in the data set used for training the model to differentiate between BK and FK, and subsequently affected the model’s accuracy [15]. Fourth, selection bias could occur in a referral medical center where a proportion of patients have received medical treatment before presentation; treatments could alter lesion features and affect the model’s learning process and performance. Fifth, we did not perform patient matching between the training, validation, and testing groups; thus, differences in clinical characteristics might also have affected the performance of the model. Sixth, we did not integrate pertinent clinical information such as risk factors for infectious keratitis, which are indispensable for clinical diagnosis. Finally, the model’s function lies in assisting in the differentiation of FK from BK, and we did not subclassify the dataset to different pathogens, which may have different clinical characteristics. Viral and parasitic keratitis were not included in this study, either. In clinical practice, cultures remain crucial for final species identification.

## 5. Conclusions

In conclusion, we developed a deep learning model for differentiating between FK and BK. The accuracy of the model was better than that previously reported for both general ophthalmologists and corneal specialists. Owing to the high accessibility of corneal images, we anticipate that the inclusion of more images would help deep learning models become a robust tool for aiding in early FK diagnosis.

## Figures and Tables

**Table 1:** Distribution of patients in the training, validation, and testing datasets.

Characteristics	Total (*n* = 580)	Training (*n* = 388)	Validation (*n* = 96)	Testing (*n* = 96)
Male, *n* (%)	420 (72.4)	278 (71.6)	74 (77.1)	68 (70.8)
Age, years ^1^	55.4 ± 20.2	53.6 ± 19.7	62.1 ± 20.0	55.8 ± 21.2
Patients, *n* (%)/Photos, *n*				
Bacteria	346 (59.7)/824	231(39.8)/562	60 (10.3)/134	55 (9.5)/128
Fungus	234 (40.3)/506	157(27.1)/342	36 (6.2)/78	41 (7.1)/86

^1^ Continuous variables presented as the mean ± standard deviation, *n*: number.

**Table 2:** Performance of different deep learning models.

Model	Validation	Testing	Average Accuracy	BK	FK	Sensitivity	Specificity	PPV	NPV
						(95% Confidence Interval)			
DenseNet121	79.7	77.9	78.8	85.0	59.6	59.7 (45.8–72.4)	85.0 (78.2–90.4)	60.7 (49.9–70.6)	84.5 (79.7–88.2)
DenseNet161	77.5	79.7	78.6	87.3	65.8	65.8 (41.5–65.8)	87.3 (86.0–95.3)	74.0 (65.1–82.9)	82.4 (74.9–82.4)
DenseNet169	83.6	75.0	79.3	79.6	63.2	63.2 (49.34–75.6)	79.6 (72.2–85.8)	54.6 (45.2–63.6)	84.8 (79.7–88.8)
DenseNet201	78.0	78.9	78.4	87.8	56.1	56.1 (42.4–69.3)	87.8 (81.3–92.6)	64.0 (52.1–74.4)	83.8 (79.3–87.4)
EfficientNetB3	77.8	74.3	76.1	85.2	58.1	58.1 (47.0–68.7)	85.2 (77.8–90.8)	72.5 (62.6–80.5)	75.2 (70.0–79.7)
InceptionV3	79.7	78.0	78.9	89.1	61.6	61.6 (50.5–71.9)	89.1 (82.3–93.9)	79.1 (69.2–86.5)	77.6 (72.4–82.0)
ResNet101	79.2	80.9	80.0	93.2	49.1	49.1 (35.6–62.7)	93.2 (87.9–96.7)	73.7 (59.3–84.3)	82.5 (78.5–86.0)
ResNet50	78.2	76.5	77.3	95.9	26.3	26.3 (15.5–39.7)	95.9 (91.3–98.5)	71.4 (50.5–86.0)	77.1 (74.1–79.7)

BK—bacterial keratitis; FK—fungal keratitis; PPV—positive predictive value; NPV—negative predictive value.

## Data Availability

The data presented in this study are available upon request. The data are not publicly available due to the data security policy of Chang Gung Memorial Hospital, Taiwan.

## Supplementary Materials

The following is available online at https://www.mdpi.com/article/10.3390/diagnostics11071246/s1, Figure S1: Examples of cropped corneal images processed through augmentation, including (A) brightness adjustment, (B) saturation adjustment, (C) contrast adjustment, (D) horizontal flipping, and (E) rotation.
