# Supplementary material for: Using Slit-Lamp Images for Deep Learning-Based Identification of Bacterial and Fungal Keratitis: Model Development and Validation with Different Convolutional Neural Networks
Source: Diagnostics (Basel). 2021 Jul 12;11(7):1246. doi: 10.3390/diagnostics11071246 (PMC8307675; doi:10.3390/diagnostics11071246)
Supplement: Supplementary file 1 [file diagnostics-11-01246-s001.zip › diagnostics-1238644-supplementary.pdf]

Supplementary Material

Using Slit-Lamp Images for Deep Learning-Based Identification of Bacterial and Fungal Keratitis: Model Development and Validation with Different Convolutional Neural Networks

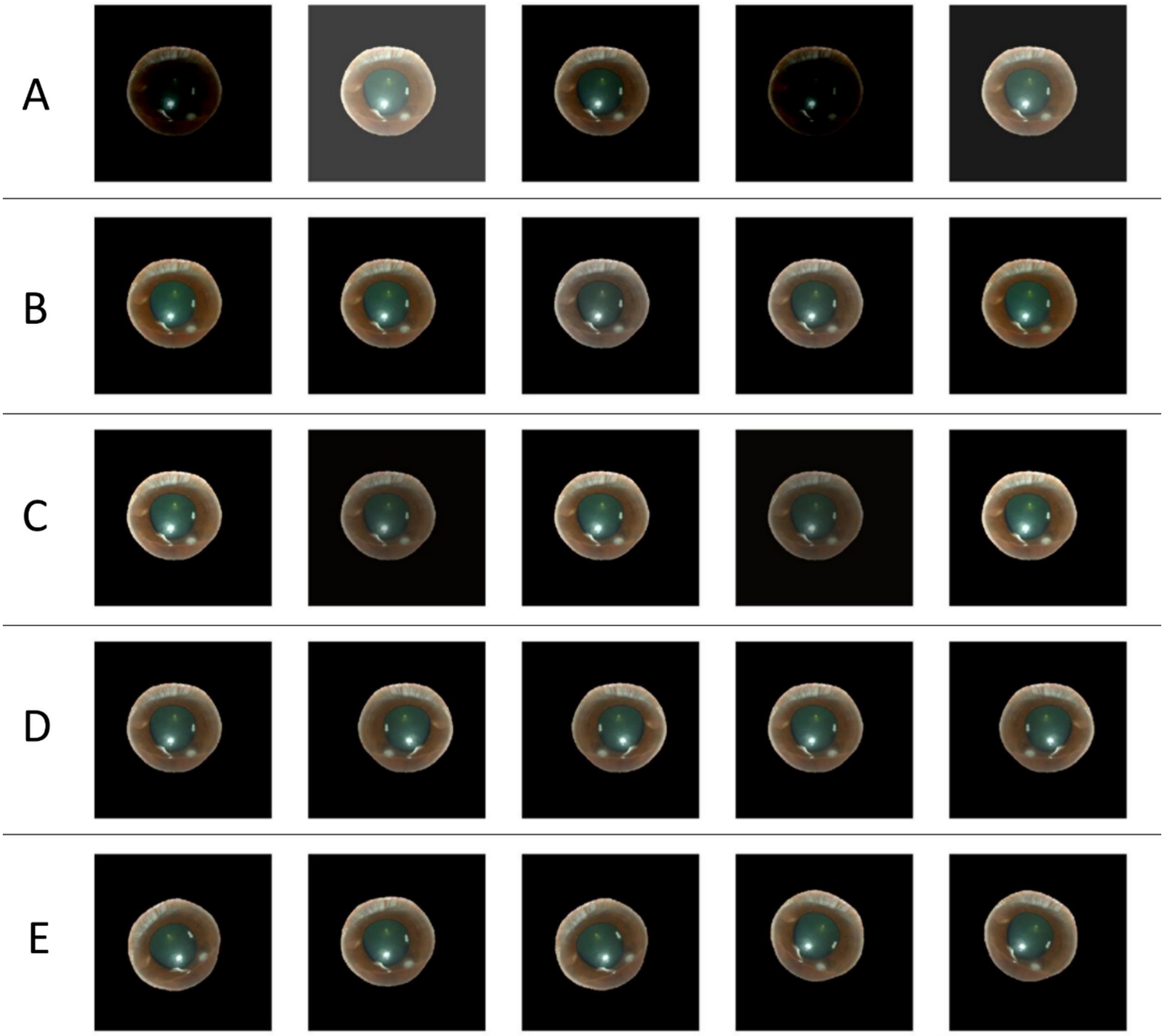

Figure S1. Examples of cropped corneal images processed through augmentation, including (**A**) brightness adjustment, (**B**) saturation adjustment, (**C**) contrast adjustment, (**D**) horizontal flipping, and (**E**) rotation.
